# An Osteolytic Metastasis of Humerus from an Asymptomatic Squamous Cell Carcinoma of Lung: A Rare Clinical Entity

**DOI:** 10.1155/2014/636017

**Published:** 2014-08-12

**Authors:** Anirban Das, Sudipta Pandit, Sibes k. Das, Sumitra Basuthakur, Somnath Das

**Affiliations:** Department of Pulmonary Medicine, Medical College, 88 College Street, Kolkata, West Bengal 700 073, India

## Abstract

Advanced lung cancer is complicated by skeletal metastases either due to direct extension from adjacent primaries or, more commonly, due to haematogenous dissemination of neoplastic cells. Lumber spine is the most common site for bony metastases in bronchogenic carcinoma. Proximal lone bones, especially humerus, are unusual sites for metastases from lung primaries. Small cell and large cell varieties of lung cancer are most commonly associated with skeletal dissemination. It is also unusual that an asymptomatic squamous cell carcinoma of lung presents with painful, soft tissue swelling with osteolytic metastasis of humerus which is reported in our case. Systemic cytotoxic chemotherapy, local palliative radiotherapy, adequate analgesia, and internal fixation of the affected long bone are different modalities of treatment in this advanced stage of disease. But the prognosis is definitely poor in this stage IV disease.

## 1. Introduction

The skeleton is a common site for metastases from epithelial tumours. Most common malignancies which present with bone metastases are carcinomas of prostate, breast, and lung [1]. Approximately one-third of the patients with bronchogenic carcinoma present with symptoms due to extrathoracic metastases [2]. In lung cancers, axial skeleton is more commonly involved than extremities [3]. Spine, ribs, pelvis, skull, and proximal long bones like femur or humerus are the bony sites for the metastases of lung cancers [3]. Thoracolumbar vertebrae are most common site for skeletal metastases in lung cancers [4]. A very few reports of metastasis to humerus in bronchogenic carcinoma are available in the literature. Here we report a rare case of bronchogenic carcinoma metastasizing to humerus and, surprisingly, the patient presented with a painful swelling of the left arm without any respiratory symptom.

## 2. Case Report

A fifty-five-year-old normotensive, nondiabetic, male smoker presented with progressively increasing soft tissue swelling in the left upper arm with intractable pain which was increasing at night for last 3 months. He also complained of weakness of left upper limb and difficulty to move the part of the limb distal to the swelling. There was history of significant weight loss, loss of appetite, and extreme fatigue, but no fever. There was no respiratory symptom or any history of contact with the patient with smear positive pulmonary tuberculosis.

General examination of the patient revealed anaemia and clubbing but no superficial lymphadenopathy. His axillary temperature was 37°C, respiratory rate 16 breaths/minute, pulse rate 84 beats/minute, and blood pressure 110/70 mmHg. Systemic examination revealed no abnormality except a tender soft tissue swelling located in the midhumerus of left side, firm in consistency, irregular in shape, and 7.5 cm × 5 cm in size with indistinct margins. Skin overlying the swelling was reddened, warm, edematous, and nodular with prominence of superficial veins but had no discharging sinus. Movements of the shoulder joints were normal. Movement of the part of the limb distal to the swelling was restricted. Biceps, triceps, and supinator jerks were absent in left side. But there was no sensory loss.

**Figure 1:**
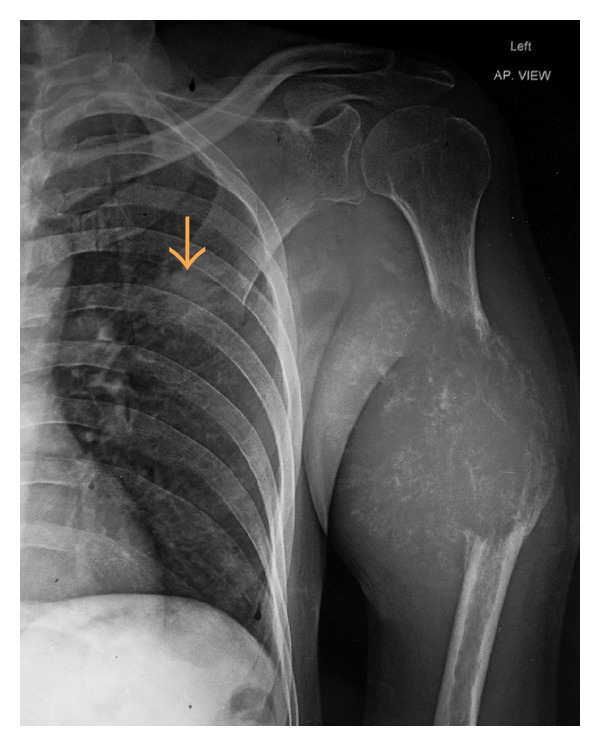
CXR-PA view showing osteolytic lesion in left midhumerus and a spiculated nodule in left midzone.

**Figure 2:**
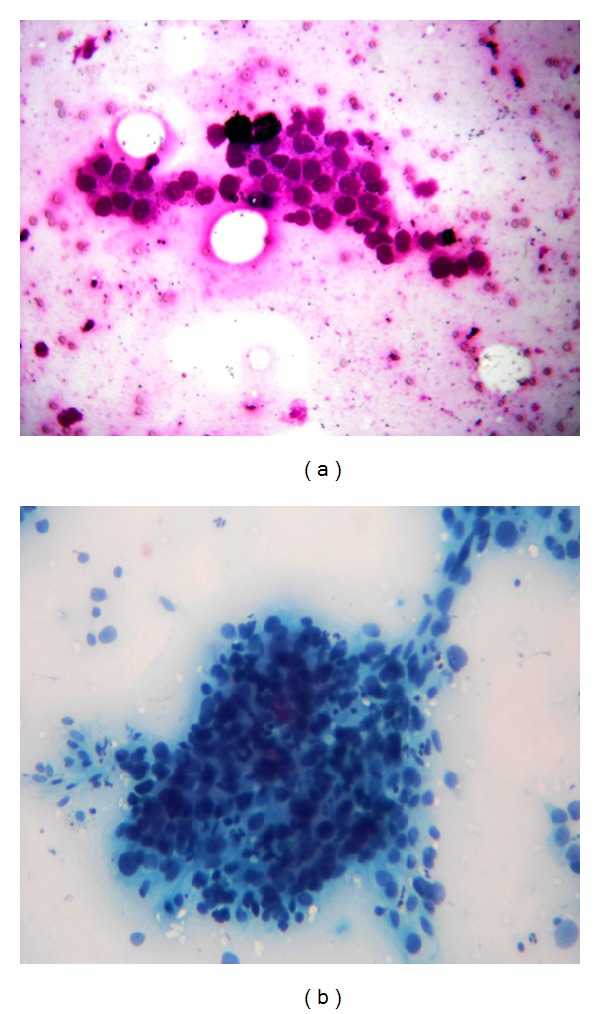
(a) Microphotograph of FNAC of osteolytic lesion of left humerus showing metastatic squamous cell carcinoma (MGG stain, 10x). (b) Microphotograph of FNAC of left lung nodule showing squamous cell carcinoma (MGG stain, 10x).

**Figure 3:**
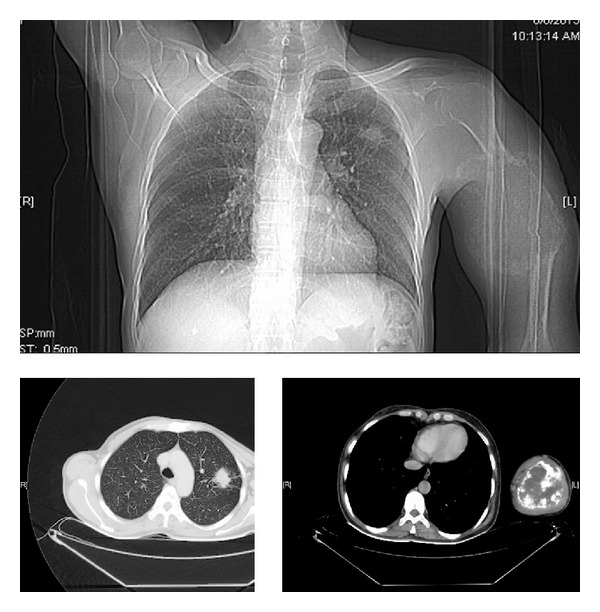
CECT thorax showing a spiculated nodule in the left upper lobe with osteolytic lesion in middle of the humerus on the left side.

**Figure 4:**
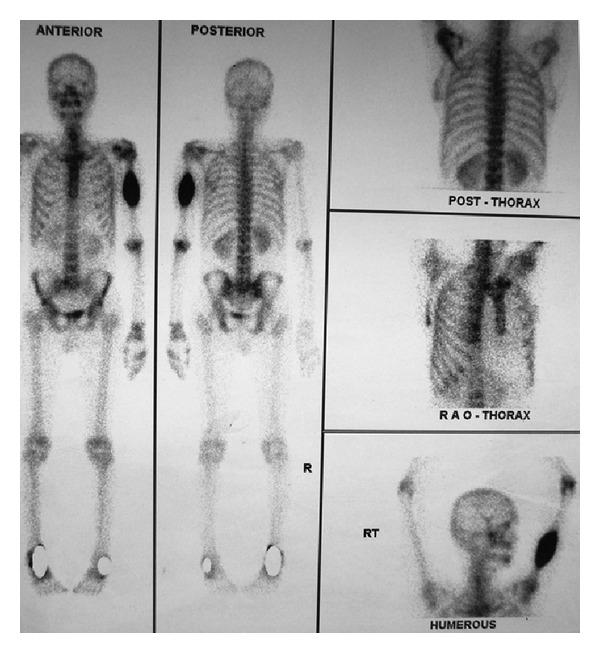
^99m^TcRadionuclide bone scan showing an increased uptake of radiotracer over the midhumerus of left side, suggestive of metastatic bony lesion.

**Figure 5:**
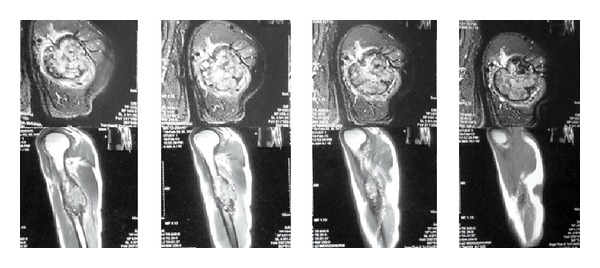
MRI of left upper extremity showing osteolytic lesion in left humerus with marrow edema and soft tissue infiltration.

**Figure 6:**
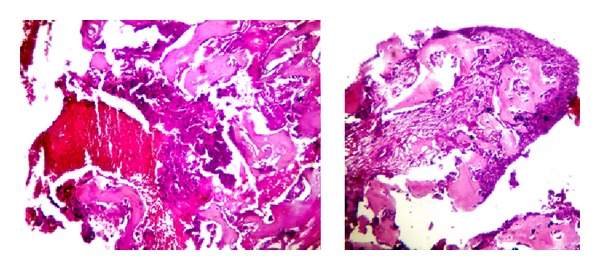
Microphotograph of HPE of resected specimen of left humerus showing metastatic squamous cell carcinoma (H&E stain, 10x).

Complete hemogram and blood biochemistry including serum calcium (9.1 mg/dL) and alkaline phosphatase were normal. Chest X-ray (CXR) posteroanterior (P.A.) view showed a spiculated nodule in the left midzone with an osteolytic lesion in middle of the left humerus (Figure 1). Fine needle aspiration cytology (FNAC) of the osteolytic lesion revealed sheets, clumps, and dense malignant cells having hyperchromatic, pleomorphic nuclei with inconspicuous nucleoli and squamoid differentiation at places on the hemorrhagic background. Few cells showed individual keratinization, suggestive of metastatic squamous cell carcinoma (Figure 2(a)). Contrast enhanced computed tomography (CECT) scan of thorax showed a spiculated nodule in left upper lobe with osteolytic lesion in the midhumerus on the left side (Figure 3). CT-guided FNAC of the left lung nodule showed clusters of malignant epithelial cells with nuclear pleomorphism, hyperchromasia, distinct nucleoli, moderate amount of cytoplasm, and distinct cell boundary, suggestive of nonsmall cell carcinoma and squamous cell variety. (Figure 2(b)). Ultrasound of the abdomen revealed no abnormality. ^99m^TcRadionuclide bone scan revealed an increased uptake of radiotracer over the midhumerus on left side only, suggestive of metastatic bony lesion to left humerus (Figure 4). So, the final diagnosis was squamous cell carcinoma of upper lobe of left lung with osteolytic metastasis to left humerus, that is, stage IV disease of bronchogenic carcinoma. Palliation of the symptoms was the only option. With consultation of the radiotherapy department of our institution, palliative radiotherapy (total dose: 30 Gy in 10 fractions) was given to the osteolytic lesion of the left midhumerus with an aim to relieve the pain and reduce the size of the lesion. First cycle of chemotherapy comprising of cisplatin + etoposide was given intravenously following radiotherapy. Although chemoradiotherapy was a very good option for palliation of the malignant bone pain, in our patient, size of the primary lung tumour was gradually increasing (as evidenced by serial CXRs) and pain of the osteolytic lesion of left humerus was not relieved, though the size of the lesion reduced marginally. As a whole, therapeutic benefits on primary and metastatic tumours were very poor, probably due to squamous cell histology which is a chemo- and radiotherapy resistant variant of lung cancer. On the other hand, the part of the limb distal to the metastatic lesion was totally nonfunctioning. This is why below shoulder amputation of the left upper limb was planned. Preoperative magnetic resonance imaging (MRI) of left upper extremity showed destructive and expansile osteolytic lesion in the junction of upper and midthird of the shaft of the left humerus, marrow edema, and surrounding soft tissue infiltration (Figure 5). After first cycle of chemotherapy amputation of upper limb was done in the department of orthopaedics, and histopathological examination of resected specimen showed metastatic squamous cell carcinoma of the bone (Figure 6). He succumbed to his illness after second cycle of chemotherapy.

## 3. Discussion

Clinical presentations of bronchogenic carcinoma are variable and of four types. The majority of patients present with new onset respiratory symptom or worsening of preexisting respiratory state (cough, hemoptysis, postobstructive pneumonia, hoarseness of voice, superior vena caval obstruction, atelectasis, etc.). A very few patients have no respiratory symptoms and an opacity on chest radiograph is detected incidentally. A third group develops nonspecific symptoms of malignancy, like malaise, anorexia, and weight loss or symptoms due to paraneoplastic syndrome. The last group presents with symptoms due to distant metastasis (bone pain, focal neurological deficits, cranial nerve palsy, symptoms due to raised intracranial tension, jaundice, abdominal pain, lymphadenopathy, metastatic nodules in contralateral lung, pleural effusion, etc.) with or without pulmonary symptoms [2]. Asymptomatic adrenal metastases or metastases to skin or skeletal muscles are seen as atypical presentations of lung cancers. Hence, this group with stage IV diseases has poor prognosis. When the patients present with extrathoracic symptoms with no respiratory manifestation, as occurring in our case, there is delay in the diagnosis, even misdiagnosis, and survival of the patients is further compromised.

In our case, the patient initially presented to orthopaedic department for the painful swelling in proximal humerus. Later, we detected the small, irregular primary tumour in left lung on chest radiograph during routine evaluation for the nature of the bony tumour, whether it was secondary or primary. In this scenario, a question was raised: which one was secondary? Is it from the humerus to lung or lung to humerus? Initially, it was thought that possibility of first condition was high, as painful swelling of the humerus was predominant manifestation, and the lung lesion was solitary, asymptomatic, and very small. But the irregular margin of the lung lesion and solitary number go against the possibility of lung metastasis. Usually pulmonary metastases are multiple, round in shape with very smooth margin, although solitary pulmonary metastasis is not unusual. FNAC of the lung mass and the swelling of the left humerus solved the problem, and final tissue diagnosis was squamous cell carcinoma of left lung with metastasis to left humerus. Absence of respiratory symptoms delayed the diagnosis in our case. But the age of the patient and history of heavy smoking raised the suspicion of primary lung malignancy in this setup. Due to overlapping histological characteristics it is sometimes impossible to differentiate between primary and metastatic lung cancer. Immunohistochemistry stain may be helpful in this situation. Cytokeratin 7 is useful for differentiation between adenocarcinoma of lung colon cancer metastasis which stains cytokeratin 20 [5]. With the advent of gene expression arrays and proteomic classification of tumours, molecular classification is an emerging tool to assist in determining whether a lung nodule is primary or secondary [6]. Another important message from this case is that, in any case of painful bone tumour, possibility of metastatic bone disease is much more than primary, because secondary tumours of bone are far more common than primaries. Small cell carcinoma of lung may present with metastatic manifestations with a small, asymptomatic lung primary, but it is very uncommon in squamous cell variety. Our case was a unique one in this respect also.

Bone metastases are of three types: osteolytic (associated with increased osteoclast activity and hypercalcaemia), osteoblastic (associated with increased activity of osteoblasts and new bone formation with raised serum alkaline phosphatase), and mixed [7]. Skeletal metastases in lung cancers are predominantly osteolytic; purely osteolytic lesion is seen only in multiple myeloma [8]. On the other hand, purely osteoblastic metastases are uncommon. Regardless of osteolytic or osteoblastic phenotype of bone metastases, osteoclastic proliferation and hypertrophy is present [9]. Bone pain is the main presentation of skeletal metastases from lung cancers. However, pathological fractures, bony swelling with soft tissue invasion, and erosion of the bones are other manifestations. Plain X-ray is adequate for detection of osteolytic metastases. However an osteolytic metastasis is not detected on conventional X-ray until there is a 30–50% loss of bone [10]. Radionuclide (^99m^Tc-methylene diphosphonate) bone scans (bone scintigraphy) show increased uptake of radioisotope due to increased osteoblastic activity and blood flow at the site of skeletal metastases [11]. Computed tomography delineates the anatomical details of the bone metastases better than plain X-ray. Magnetic resonance imaging (MRI) is superior to bone scintigraphy with respect to sensitivity, specificity, and the extent of metastatic involvement [10]. MRI is also useful for detection of invasion of adjacent soft tissue and vascular invasion and especially useful to exclude cord compression in vertebral metastases [12]. [18F]-fluorodeoxyglucose positron emission tomography (FDG-PET) is another promising method for detection of bone metastases but is less sensitive than MRI in detection of osteal metastases [10]. FNAC is used to confirm the diagnosis of skeletal metastases with 100% cytodiagnostic accuracy [12]. A biopsy should be done to confirm the histopathological type of metastatic carcinomas of bones.

The patient had stage IV lung cancer with poor prognosis. Palliative local radiotherapy may be given to the painful metastases of the humerus to relieve the pain (as it was refractory to nonopioid and opioid analgesics) and also to reduce the size of the lesion with intent to unite the pathological fracture of the humerus with the help of internal fixation [13, 14]. Cytotoxic chemotherapy consisting of cisplatin and gemcitabine may be given for palliation. Bisphosphonates like zoledronic acid may be used to treat hypercalcaemia [15]. Curative resection of both the tumours in a case of primary lung cancer with a solitary metastasis to adrenal gland or brain is very much successful [16], but it may not be applicable in other solitary metastases like bone, as in our case.
